# Sex Pheromone Receptor Specificity in the European Corn Borer Moth, *Ostrinia nubilalis*

**DOI:** 10.1371/journal.pone.0008685

**Published:** 2010-01-13

**Authors:** Kevin W. Wanner, Andrew S. Nichols, Jean E. Allen, Peggy L. Bunger, Stephen F. Garczynski, Charles E. Linn, Hugh M. Robertson, Charles W. Luetje

**Affiliations:** 1 Department of Plant Sciences and Plant Pathology, Montana State University, Bozeman, Montana, United States of America; 2 Department of Molecular and Cellular Pharmacology, Miller School of Medicine, University of Miami, Miami, Florida, United States of America; 3 Yakima Agricultural Research Laboratory, Agricultural Research Service, United States Department of Agriculture, Wapato, Washington, United States of America; 4 Department of Entomology, Barton Laboratory, New York State Agricultural Experiment Station, Cornell University, Geneva, New York, United States of America; 5 Department of Entomology, University of Illinois at Urbana-Champaign, Urbana, Illinois, United States of America; University of California Davis, United States of America

## Abstract

**Background:**

The European corn borer (ECB), *Ostrinia nubilalis* (Hubner), exists as two separate sex pheromone races. ECB(Z) females produce a 97∶3 blend of Z11- and E11-tetradecenyl acetate whereas ECB(E) females produce an opposite 1∶99 ratio of the Z and E isomers. Males of each race respond specifically to their conspecific female's blend. A closely related species, the Asian corn borer (ACB), *O. furnacalis*, uses a 3∶2 blend of Z12- and E12-tetradecenyl acetate, and is believed to have evolved from an ECB-like ancestor. To further knowledge of the molecular mechanisms of pheromone detection and its evolution among closely related species we identified and characterized sex pheromone receptors from ECB(Z).

**Methodology:**

Homology-dependent (degenerate PCR primers designed to conserved amino acid motifs) and homology-independent (pyrophosphate sequencing of antennal cDNA) approaches were used to identify candidate sex pheromone transcripts. Expression in male and female antennae was assayed by quantitative real-time PCR. Two-electrode voltage clamp electrophysiology was used to functionally characterize candidate receptors expressed in *Xenopus* oocytes.

**Conclusion:**

We characterized five sex pheromone receptors, OnOrs1 and 3–6. Their transcripts were 14–100 times more abundant in male compared to female antennae. OnOr6 was highly selective for Z11-tetradecenyl acetate (EC_50_ = 0.86±0.27 µM) and was at least three orders of magnitude less responsive to E11-tetradecenyl acetate. Surprisingly, OnOr1, 3 and 5 responded to all four pheromones tested (Z11- and E11-tetradecenyl acetate, and Z12- and E12-tetradecenyl acetate) and to Z9-tetradecenyl acetate, a behavioral antagonist. OnOr1 was selective for E12-tetradecenyl acetate based on an efficacy that was at least 5-fold greater compared to the other four components. This combination of specifically- and broadly-responsive pheromone receptors corresponds to published results of sensory neuron activity *in vivo*. Receptors broadly-responsive to a class of pheromone components may provide a mechanism for variation in the male moth response that enables population level shifts in pheromone blend use.

## Introduction

Sex pheromone communication between male and female moths is believed to have contributed to their extensive speciation [1]. More than 98% of the 150,000 described extant species of Lepidoptera belong to the Ditrysia, a monophyletic lineage that evolved during the last 110 million years [2]. Female moths produce and release a mixture of related fatty acid derivatives from their pheromone gland to which males respond from long distances. In many cases, subtle changes in carbon chain length, double bond location and isomer blend differentiate the pheromones of closely related species [3]. While a variety of mating systems have evolved in the Lepidoptera, female release of pheromone is a predominant ancestral trait [4]. One long standing question has been the origin and mechanism of the variation in detection that enables the evolution of new pheromone blends.

The European corn borer (ECB), *Ostrinia nubilalis* (Hubner), has provided a model system to study the evolution of sex pheromones among closely related races and species. Most of the 20 species in the genus *Ostrinia* use varying ratios of Z11- and E11-tetradecenyl acetate (Z11- and E11-14:OAc) as the two main components of their pheromone blend [5]–[7]. An introduced pest from Europe, the ECB was first detected in North America in 1917 and exists as two different pheromone races [8]. Males of the Z-race are attracted to a 97∶3 blend of Z11- and E11–14:OAc whereas ECB(E) males are attracted to a 1∶99 blend of the Z and E isomers [9]–[10]. The closely related Asian corn borer (ACB), *O. furnacalis*, is unique in this genus, having evolved to use a pheromone blend with a shift in the location of the double bond, Z12- and E12-tetradecenyl acetate (Z12- and E12-14:OAc) [11]. Mating isolation between the Z- and E-races of ECB is controlled by a few major genetic loci, including *pher* and *resp*, controlling female blend production and male response, respectively [12]–[15]. Desaturase enzymes in the female moth pheromone gland introduce double bonds at specific locations along the hydrocarbon chain. The recruitment of a novel Δ14 desaturase into the pheromone biosynthesis pathway of an ancestor of the ACB led to a novel pheromone blend (Z12- and E12–14:OAc) contributing to the divergence of this species from the ECB [16].

Male moths have evolved to detect female-produced sex pheromones with great sensitivity and specificity over a wide range of concentrations [17]. A majority of the olfactory neurons on male antennae, housed within long trichoid sensilla, specifically respond to components of the female sex pheromone. The sex pheromones are detected by odorant receptors (Ors) expressed on the dendrites of the olfactory neurons [18]–[19]. The trichoid sensilla on male ECB and ACB antennae typically house three different olfactory neurons that can be differentiated by the amplitude of their electrophysiological response spikes. For ECB(E) males, a large-spiking neuron responds to the main pheromone component, a small-spiking neuron responds to the minor component, and an intermediate-spiking neuron responds to Z9-tetradecenyl acetate (Z9-14:OAc) [20]–[24]. The olfactory pathway responding to Z9-14:OAc antagonizes responses to the attractive pheromone pathway and prevents upwind flight to similar sex pheromone blends that include Z9-14:OAc [6].

Insect Ors are a family of chemoreceptors (Cr) that function as ligand-gated ion channels [25]–[27]. A highly conserved Or termed 83b in *Drosophila melanogaster* and its ortholog in other insect species acts as a chaperone and dimerization partner for other Ors that impart ligand specificity [28]. Together Or83b+Or_x_ form a ligand-gated ion channel. Approximately 10% of the expected 60–70 Or genes encoded in moth genomes form a distinct phylogenetic subfamily that appears to be dedicated to sex pheromone detection [18]–[19]. Seven silkworm (*Bombyx mori*) and six tobacco budworm (*Heliothis virescens*) Ors belong to this subfamily. All but two are expressed at higher levels in male antennae [29]–[30] and four respond to their respective sex pheromone components *in vitro* [18], [31].

The behavioral response of male insects to sex pheromone can be closely linked to the activity of the peripheral olfactory neurons. Transgenic fruit flies expressing the silkworm pheromone receptor BmOr1 [18] in place of their sex and aggregation pheromone receptor DmOr67d [32] are attracted to the silkworm pheromone bombykol rather than their own pheromone vaccenyl acetate [33]. Activation of the sex- and aggregation-specific olfactory pathway results in behavioral attraction independent of the actual signal. The neurological pathway of sex pheromone sensitive olfactory neurons and their projection to the antennal lobe was recently compared between ECB(Z) and ECB(E) males. In each case, the axons of the large-spiking neurons that respond to the main pheromone component, Z11-14:OAc for ECB(Z) and E11–14:OAc for ECB(E), projected to the same macroglomerulus in the male antennal lobe [34]. The authors concluded that the major genetic locus that controls the altered olfactory response between the Z and E races did not result in a rewiring of the olfactory neurons, rather, the mechanisms must be located at the periphery. Ors belonging to the sex pheromone receptor subfamily are excellent candidates because the activity of an olfactory neuron often parallels the response spectrum of the Or that it expresses [35]. Here we employed a functional genomics approach to identify and characterize five sex pheromone receptors from ECB(Z) moths to further explore peripheral mechanisms contributing to the evolution of sex pheromone detection.

## Results

### Five Candidate Sex Pheromone Receptors Identified from ECB(Z)

Two complementary approaches were used to identify the greatest possible number of candidate sex pheromone receptors in the absence of whole genome sequencing. First, degenerate PCR primers were designed to match a conserved amino acid motif in the carboxy(C)-terminus of known Lepidoptera sex pheromone receptors, (I/L/V)PW(E/D)(Y/F/C/H/A)M(D/N)(T/V/K/I/N). Using these degenerate primers, the C-terminus of five Or transcripts with amino acid homology to the Lepidoptera sex pheromone receptor subfamily were identified by 3′ Rapid Amplification of cDNA Ends (RACE) reactions (GeneBank accession numbers FJ385011 - FJ385015).

In a second approach, an EST library was created by high-throughput pyrophosphate sequencing of antennal cDNA. Seven partial cDNA sequences with amino acid homology to known Lepidoptera sex pheromone receptors were identified by tBLASTn searches of the assembled contigs (Text S1). The seven contigs varied from 178 to 1124 nucleotides (nt) in length, and were assembled from a minimum of 6 sequence reads to a maximum of 198 reads (Table S1). OnOr2, the ortholog of DmOr83b that acts as a chaperone and partner for most Ors, was represented by two contigs (Table S1) of 1032 and 178 nt (62 and 6 reads, respectively). All cDNAs were partial sequences, 3′ and 5′ RACE was required to clone and sequence the complete open reading frames (ORFs).

As a result, the combined approaches yielded 5 unique cDNAs, *OnOr1* and *3–6* (GenBank Accession numbers GQ844876-GQ844881) that were cloned using primers designed from the RACE sequences. *OnOr1* and *OnOr 3–6* encode proteins ranging from 421 to 425 amino acids in length including motifs characteristic of the insect Or family (such as the conserved C-terminal serine and tyrosine residues,36; Figure S1). All five Ors have BLASTp similarity to lepidopteran sex pheromone receptors that have been functionally characterized. OnOr 1 and 6 are 36% and 41% identical to *Plutella xylostella* Or1 [37]; OnOr3 is 36% identical to *Diaphania indica* Or1 [37]; and, OnOr 4 and 5 are 63% and 99% identical to a sex pheromone receptor recently characterized from *O. nubilalis* [7].

ESTs representing *OnOr 4* and *5* were identified by 3′ RACE with degenerate primers but were not represented by pyrosequencing contigs. Conversely, ESTs representing *OnOr6* were abundantly represented by pyrosequencing contigs but were not amplified using degenerate primers. These results illustrate the benefit of using two complementary approaches to identify candidate pheromone receptors, one dependent on sequence homology and the other independent of sequence homology, but dependent on adequate expression levels.

It was uncertain whether the pyrosequencing approach would provide sufficient sequence coverage of rare transcripts to assemble contigs that could be detected by tBLASTn searches. The full length nucleotide sequences of *OnOrs 1–6* used as queries for BLASTn searches yielded only three new contigs (Figure S2). These contigs were not detected in our original tBLASTn searches because they contained intron or 3′UTR sequence and less than 120 nt of coding sequence.

### *OnOrs 1* and *3–6* Are Expressed at Higher Levels in Male Antennae

**Figure 1:**
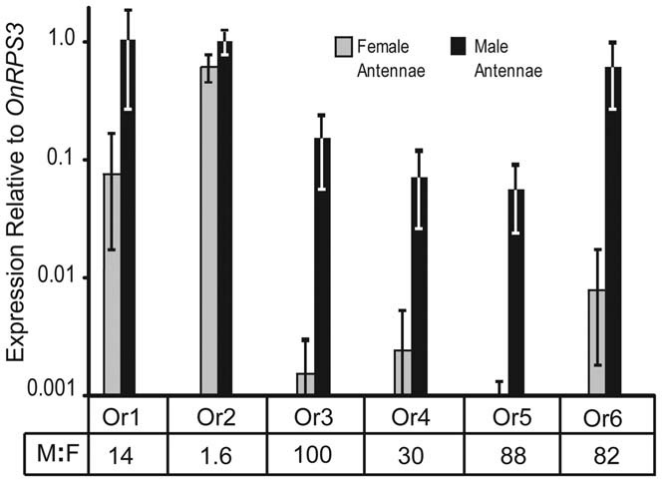
Male-biased expression of five ECB(Z) sex pheromone receptor genes. Ratios of male to female expression (M:F) are presented below each bar. Gene expression, determined by real-time quantitative PCR with SYBR green, is reported relative to the reference gene OnRPS3 on a logarithmic scale. Expression values are presented as averages (with standard error bars) of four biological replicates and three nested technical replicates. Sex-biased expression is supported by nested ANOVA analyses of the normalized CT values, P = 0.03, 0.04, 0.001, 0.06, 0.001 and 0.003, OnOrs1-6 respectively.

Expression levels of *OnOrs 1* and *3–6*, averaged from four biological replications, were determined by quantitative real-time PCR (qPCR). The transcripts of all five candidate pheromone receptors were expressed at higher levels in male antennae, ranging from 14 to 100 times higher compared to female antennae (Figure 1). *OnOr2* was highly expressed at levels comparable to the reference gene *ribosomal protein S3* (*OnRPS3*) and only 1.6 times higher in the male antennae. *OnOr 1* and *6* transcripts were detected at similarly high levels, whereas the transcripts of *OnOrs3–5* were approximately an order of magnitude less abundant (Figure 1). In general, *OnOrs1-6* were not expressed at significant levels in other tissues such as legs, abdomen and mouthparts (Figure S2). *OnOr1* expression in female but not male mouthparts, and *OnOr3* expression in male but not female abdomens, may be two interesting exceptions (Figure S2). Low level signal, more than two orders of magnitude below that of the reference gene *OnRPS3*, can result from non-specific PCR amplification that is detected by the SYBR green dye or by genomic DNA contaminating the RNA template. In addition to removing DNA from the RNA template by enzyme digestion, false expression signal from contaminating DNA was assessed by including RNA that was not reverse transcribed. These negative controls did not produce signals of expression confirming the purity of the RNA template. In addition, several primer sets spanned an intron, and the absence of larger-sized amplicon that would result from genomic DNA template was confirmed by gel electrophoresis of the PCR product and by its melting point curve.

### Specific and Broad Responses of Different ECB Sex Pheromone Receptors

**Figure 2:**
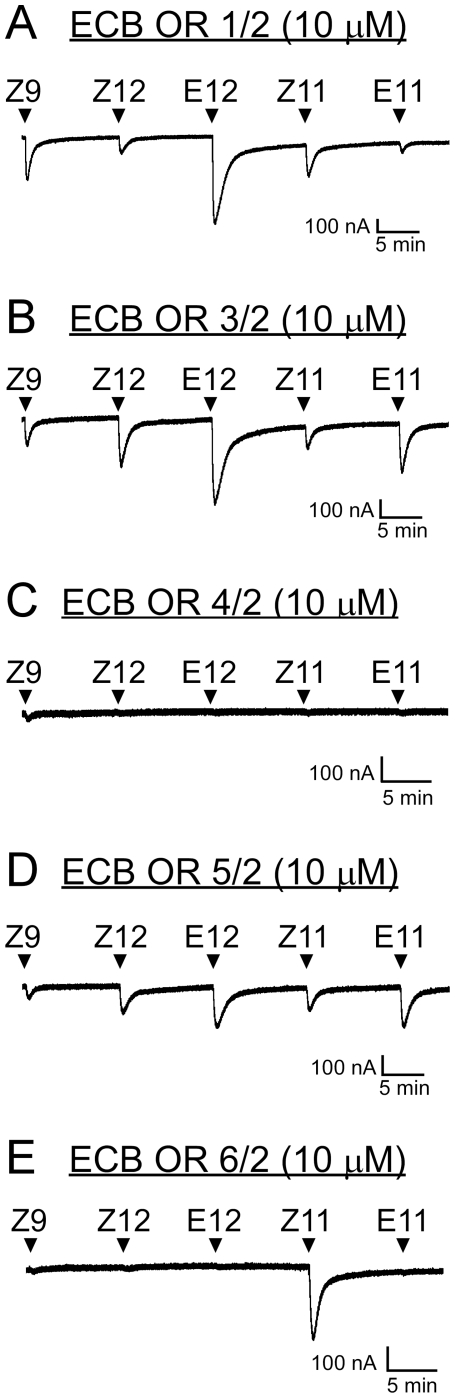
Functional screen of candidate ECB(Z) pheromone receptors. Oocytes expressing OnOr2 and either OnOr1 (A), OnOr3 (B), OnOr4 (C), OnOr5, (D) or OnOr6 (E) were challenged with 20 sec applications (arrowheads) of various ECB and ACB pheromones (at 10 µM): Z9–14:OAc (Z9), Z12–14:OAc (Z12), E12–14:OAc (E12), Z11–14:OAc (Z11), and E11–14:OAc (E11). Each application was separated by 10 min washing in ND96 (4.6 ml/min). Pheromone-induced currents were measure by two-electrode voltage clamp electrophysiology.

Each of the five candidate ECB(Z) receptors was co-expressed in *Xenopus* oocytes with the obligatory functional partner OnOr2, and screened for responsiveness to a panel of ECB and ACB pheromone components (Z12–14:OAc, E12–14:OAc, Z11–14:OAc, and E11–14:OAc), and the antagonist Z9–14:OAc, at a 10 µM concentration (Figure 2). OnOr4/2 failed to be activated by any of the components tested, with the exception of a very slight response to the antagonist Z9–14:OAc (Figure 2C). Increasing the concentration of Z9–14:OAc to 300 µM did not increase the response amplitude (unpublished results), suggesting that OnOr4/2 may not be robustly expressed in our assay system, or the receptor responds to a ligand not tested here. OnOr6/2 was specifically activated only by Z11–14:OAc (Figure 2E). Surprisingly, OnOr1/2, OnOr3/2, and OnOr5/2 responded to all five components (Figure 2A, B and D). OnOr3/2 and OnOr5/2 exhibited only slight isomer selectivity, both favoring the E isomers over the Z isomers. OnOr1/2 did not share this trend; it was more selective for E12–14:OAc over Z12–14:OAc, but surprisingly, was selective for Z11–14:OAc over E11–14:OAc.

### OnOr6 Is a Highly Specific Receptor Tuned to Z11–14:OAc

**Figure 3:**
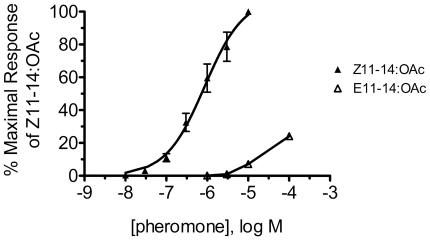
Dose-response relationships for Z11-14:OAc and E11-14:OAc activation of OnOr6/2. Pheromone-induced currents were measure by two-electrode voltage clamp electrophysiology. Refer to Table 1 for EC_50_, Hill slope, and relative efficacy values. Data is presented as means ± SEM (Z11–14:OAc, n = 4; E11–14:OAc, n = 5).

We next investigated the specificity of OnOr6/2 through a range of pheromone concentrations. Dose-response analysis revealed OnOr6/2 to be a sensitive receptor for Z11–14:OAc, with an apparent EC_50_ of 0.86±0.27 µM (mean ± SEM, n = 4) (Figure 3). Although E11–14:OAc began to elicit a receptor response at higher concentrations, approximately half of this response can be attributed to the small amount of Z11–14:OAc present in our sample of E11–14:OAc (0.1%, personal communication, Pherobank, Wageningen, The Netherlands). If the remaining response is truly due to E11–14:OAc, then OnOr6/2 is approximately 1000-fold selective for Z11–14:OAc over E11–14:OAc. These results demonstrate that OnOr6/2 is highly specific for Z11–14:OAc, exhibiting a strong degree of isomer selectivity.

### Based on Relative Efficacy, OnOr1 Responds Best to E12–14:OAc

**Figure 4:**
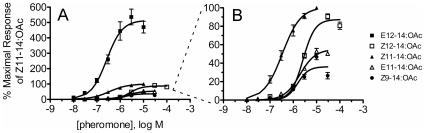
Dose-response relationships for E12–14:OAc, Z12–14:OAc, Z11–14:OAc, E11–14:OAc and Z9–14:OAc activation of OnOr1/2. Left and right graphs have different y-axis scales of the same data points. Pheromone-induced currents were measure by two-electrode voltage clamp electrophysiology. Refer to Table 1 for EC_50_, Hill slope, and relative efficacy values. Data is presented as means ± SEM (E12–14:OAc, n = 5; Z12–14:OAc, n = 6; Z11–14:OAc, n = 7; E11–14:OAc, n = 5; and Z9–14:OAc, n = 5).

Although OnOr1/2 responded to all five components, this receptor exhibited unique preferences as compared to OnOr3/2 and OnOr5/2. Therefore, dose-response analysis was performed for all five pheromones (Figure 4). While OnOr1/2 was broadly activated by the various pheromones with similar potencies, we observed a wide range of relative efficacies that may provide a mechanism for OnOr1/2 to differentiate among pheromone isomers (Table 1). Based on this analysis, we conclude that E12–14:OAc is the strongest activator of OnOr1/2.

**Table 1:** Summary data of the activation of OnOr1/2 and OnOr6/2 by ECB and ACB pheromones and the antagonist Z9–14:OAc.

OnOr1/2	EC_50_ (µM ± SEM)	Hill slope	Relative Efficacy (% response to Z11–14:OAc)
**Z9–14:OAc**	1.54±0.33	1.85	36.1±2.8
**Z12–14:OAc**	2.73±0.24	1.68	87.4±2.3
**E12–14:OAc**	0.26±0.05	1.30	515±30.9
**Z11–14:OAc**	0.34±0.05	1.10	100.7±3.9
**E11–14:OAc**	1.92±0.20	1.31	55.0±2.0
**OnOr6/2**			
**Z11–14:OAc**	0.86±0.27	0.91	100

### Phylogenetic Relationship of OnOrs1-6 within the Pheromone Receptor Subfamily

**Figure 5:**
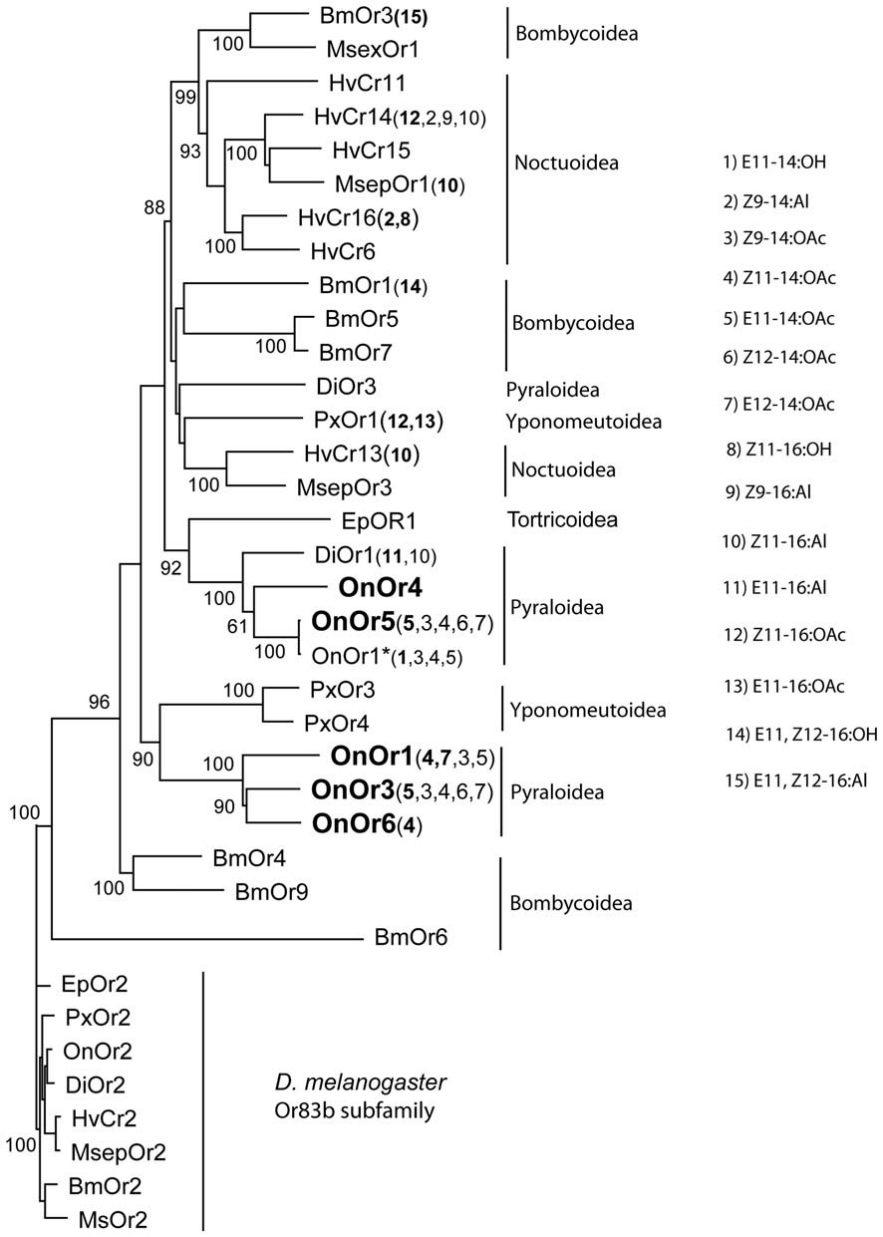
Phylogenetic relatedness of OnOrs1-6 to the Lepidoptera sex pheromone receptor subfamily, neighbor-joining (corrected distance) tree. Bootstrap values are presented as a percentage of n = 1000 replicates at significant branch points. The tree is rooted with lepidopteran orthologs of DmOr83b. The responses of receptors that have been functionally characterized are indicated by numbers corresponding to the 15 pheromone compounds listed, bolded numbers indicate the strongest response. Bm, *Bombyx mori*; Di, *Diaphania indica*; Ep, *Epiphyas postvittana*; Hv; *Heliothis virescens*; Msex, *Manduca sexta*; On, *Ostrinia nubilalis*; Px, *Plutella xylostella*; Msep, *Mythimna separata*. Superfamily taxonomies are delineated by vertical bars. ECB receptors reported in this study are bolded; OnOr1* was reported in [7] and is identical to OnOr5 in this study. Pheromone ligands: 1) E11–14:OH; E11-tetradecen-1-ol, 2) Z9–14:Al; Z9-tetradecenal, 3) Z9–14:OAc; Z9-tetradecenyl acetate, 4) Z11–14:OAc; Z11-tetradecenyl acetate, 5) E11–14:OAc; E11-tetradecenyl acetate, 6) Z12–14:OAc; Z12-tetradecenyl acetate, 7) E12–14:OAc; E12-tetradecenyl acetate, 8) Z11–16:OH; Z11-hexadecen-1-ol, 9) Z9–16:Al; Z9-hexadecenal, 10) Z11–16:Al; Z11-hexadecenal,11) E11–16:Al; E11-hexadecenal, 12) Z11–16:OAc; Z11-hexadecenyl acetate, 13) E11–16:OAc; E11-hexadecenyl acetate, 14) E10, Z12–16:OH; E10,Z12-hexadecadien-1-ol, and 15) E10, Z12–16:Al; E10,Z12-hexadecadienal.

The 28 published Or sequences from 8 different species that belong to the lepidopteran pheromone receptor subfamily group together generally at the superfamily level of taxonomy (Figure 5). OnOrs1, 3 and 6 are most related to each other and group together with two Ors from the diamondback moth, *Plutella xylostella*. OnOrs 4 and 5 group together on a separate lineage along with an Or from the light brown apple moth *Epiphyas postvittana* and an Or from the cucumber moth *Diaphania indica*. With the current representation of published sequences there is no clear relationship between pheromone receptor phylogeny and their ligand response. For example, HvCr14 and PxOr1 both respond best to Z11-16:OAc and HvCr13 and MSepOr1 both respond best to Z11-16:Al (Figure 5)[31], [37]. The receptors do not appear to be orthologous in either case.

## Discussion

Chemical communication in mating behavior is a prominent feature of moth biology that has contributed to their extensive divergence. To understand better how the molecular mechanisms of sex pheromone detection evolve we identified and characterized five sex pheromone receptors from the ECB(Z), a model example of an early stage of speciation [38]. OnOr6 was particularly interesting as it responded with high specificity and isomer selectivity to Z11–14:OAc, the main component of the ECB(Z) pheromone blend. Based on EC_50_ values, OnOr6 is at least 1000 times more responsive to Z11–14:OAc compared to E11–14:OAc (Figure 3). Importantly, these *in vitro* results correspond to *in vivo* electrophysiological recordings that found a large-spiking neuron in ECB(Z) males, and a small-spiking neuron in ECB(E) males that responded specifically to Z11–14:OAc [22]–[23]. Consequently, OnOr6(Z) should be expressed in the large-spiking neurons of ECB(Z). Its ortholog in ECB(E) males is likely expressed in the small-spiking neurons, but further research will be required to test this hypothesis.

We did not find a similar receptor that responded specifically to E11–14:OAc. An additional sex pheromone receptor that responds specifically to E11–14:OAc that was not identified by our approach might also exist. Traditionally it has been thought that male moth antennae possess olfactory neurons specifically tuned to each of the components of the female sex pheromone blend [39]–[41]. Rather, we found that the remaining pheromone receptors responded generally and more broadly to the five compounds tested. OnOr1 responded to all five compounds tested with EC50s ranging from 0.26 uM to 2.73 uM (Table 1). BmOr1 and 3, the silkworm bombykol and bombykal receptors, responded with similar sensitivities to their pheromone ligands when co-expressed with BmOr2 in *Xenopus* oocytes (EC50s 0.26 and 1.5 uM, respectively)[18]. These results are similar to recent electrophysiological data finding that the large-spiking neurons of ECB(E) males actually respond more broadly *in vivo* [22]–[23]. This neuron responded best to E11–14:OAc but it also responded to the Z11-, E12- and Z12- 14:OAc components. However, co-expression of two or more pheromone receptors in the same olfactory neuron could also explain the more broad *in vivo* responses [23].

The existence of more broadly-responsive sex-pheromone receptors *in vitro*, and pheromone-sensitive olfactory neurons *in vivo*, suggests that not all components of a pheromone blend need to be detected with high specificity. Male moths respond to the ratios of the major and minor components in a pheromone blend [22]. If behavioral attraction requires activity of both neuron types at specific ratios, behavioral specificity can be retained with one highly-specific neuron and one more generally-responding neuron. A combination of specific- and generally-responsive pheromone receptors may provide the genetic variability for males to detect and track shifts in female pheromone blend production [42].

‘Rare’ ECB and ACB males, typically representing 3–5% of the population, are less specific in their behavioral response to related pheromone blends [6]. Changes in the periphery that alter the strength or specificity of the olfactory neuron's response to specific pheromone components could account for the rare responses [22]–[24]. For example, a decrease in responsiveness of the small-spiking neuron of rare ECB(E) males to Z12–14:OAc may alter the firing ratio relative to the large-spiking neuron in a way that allows the ACB blend to mimic the ECB(E) blend [22]. The antagonism-related olfactory neuron of normal ACB males responds to Z11–14:OAc in addition to Z9–14:OAc, preventing flight of ACB males to the ECB pheromone blend. However, this response to Z11–14:OAc in the antagonism pathway is lacking in rare ACB males that fly to the ECB pheromone [24]. Amino acid polymorphisms between alleles of a more broadly tuned Or could account for subtle changes in olfactory neuron response. Such variation could also provide the genetic material for the evolution of altered detection and response to new pheromone blends [22]. OnOr1 in this study exhibited a more efficacious response to the E12–14:OAc ligand. Its ortholog in the ACB might be a candidate receptor for one of its main pheromone components, E12–14:OAc.

Alternatively, the broad *in vitro* responses measured in this study may not completely reflect their *in vivo* specificity. While the Ors are clearly one of the major determinants of olfactory neuron specificity, complexes of interacting proteins are involved in the signal transduction, including sensory neuron membrane protein 1 (SNMP1) and pheromone binding proteins (PBPs) [43]. For example, PBPs can increase physiological sensitivity to pheromone ligands. BmOr1 expressed in the empty neurons of *Drosophila* ab3 sensilla is activated by the silkworm sex pheromone bombykol. However, when co-expressed with BmPBP1, much lower concentrations of bombykol activate the BmOr1-expressing neuron [44]. PBPs may also affect the specificity of the physiological response to sex phoromone. A PBP added to an *in vitro* assay altered the specificity of a moth pheromone receptor, making its response more specific [31]. Similarly, the responses of OnOr1 and 3 characterized in this study may be more specific *in vivo* in the presence of PBPs. Also, the responses of OnOr1 and 3–6 to a larger panel of pheromone and general odors should be tested in future work. OnOr5 in this study corresponds to an ECB Or that was recently reported to respond to E11-tetradecen-1-ol, a pheromone component used by ancestral species in the genus *Ostrinia* [7].

The male moth olfactory system that responds to the female-produced sex pheromone is believed to be subject to stabilizing selection. Duplication of desaturase enzyme genes and their differential activation in the pheromone glands of female ECB and ACB moths provides a mechanism for sudden changes in the pheromone blend [45]–[46]. The origins of variation in male detection and response that enable the evolution of new sex pheromone blends has been a long-standing question [16]. To address this, the asymmetric tracking hypothesis proposed that male responses were broad enough to track changes in female production [42]. Physiological studies of the pheromone-sensitive ORNs of rare ECB males that respond to ACB pheromone provided support for this hypothesis [22]. The existence of both specifically- and broadly-responsive sex pheromone receptors may represent a molecular mechanism; however, further *in vitro* and *in vivo* experiments will be required to test this hypothesis.

### Ethics Statement

The care and use of *X. laevis* frogs in this study were approved by the University of Miami Animal Research Committee and meet the guidelines of the National Institutes of Health.

## Materials and Methods

### Insects and RNA Extraction

ECB(Z) pupae were purchased from Benzon Research (Carlisle, Pennsylvania) and provided from a colony maintained at the New York State Agricultural Experiment Station. Antennae were dissected from male and female adults within 3 days of emergence. Mouthparts, legs, and abdomens were dissected separately. All tissues were stored at −80° C. For gene expression studies antennae were collected from four batches each consisting of 35–50 male and 35–50 female moths. RNA was extracted from frozen tissue using a Dounce homogenizer and an RNeasy Mini kit (Qiagen, Valencia, CA). RNA was quantified and assayed for purity by absorbance at 260 nm, 280 nm, and 230 nm using a NanoDrop 1000 Spectrophotometer (Thermo Scientific, Waltham, MA).

### Pyrosequencing and Or EST Identification

cDNA was prepared by the University of Illinois Urbana-Champaign W.M. Keck Center for Comparative and Functional Genomics from 200 µg of pooled antennal total RNA (100 µg from male and female antennae). The cDNA was pyrosequenced using a Roche 454 GS-FLX system and the sequence reads assembled into contigs. FASTA files of the non-redundant contigs were formatted as BLAST databases and searched using a PC version of standalone BLAST. Silkworm Or sequences were used as queries in tBLASTn searches to identify EST contigs with homology to known lepidopteran sex pheromone receptors. Detailed methods and results of the EST library will be presented elsewhere.

### Or Cloning

3′ and 5′ RACE-ready cDNA was generated from male ECB antennal total RNA using the SMART RACE cDNA Amplification kit (Clontech, Mountain View, CA). Forward and reverse gene-specific primers designed from ESTs with homology to lepidopteran sex-pheromone receptors were combined with the SMART RACE primers (Invitrogen, Carlsbad, CA) to amplify PCR products. PCR reactions used the Advantage 2 Polymerase Mix (Clontech) under the following conditions: 94°C for 3 minutes, 24 cycles of 94° for 20 seconds, 68° for 6 minutes, followed by 1 cycle of 72° for 5 minutes. In some cases a second internal gene-specific reverse primer was used for nested 5′RACE. 3′ and 5′ RACE products were gel purified (Qiagen MinElute Gel Extraction Kit), cloned into the TOPO pCR2.1 vector (Invitrogen TOPO TA cloning kit) and sequenced in both directions. The resulting sequences were used to design forward and reverse primers (with restriction enzyme sites for pGEMHE) to amplify the complete ORFs of five unique Ors (*OnOrs1* & *3–6*) and the *DmOr83b* ortholog. Each TOPO clone was sequenced in both directions and the inserts subcloned into the pGEMHE vector which was subsequently sequenced in both directions. The relationships of translated Or sequences were analyzed by constructing a neighbor-joining phylogenetic tree using PAUP software [47]. Corrected distances were used to construct the tree and uncorrected distances to perform bootstrap analysis (n = 1000 replicates) as described in [48].

### Gene Expression

Genomic DNA was digested from Total RNA used for gene-expression with the TURBO DNA-free kit (Applied Biosystems, Foster City, CA). cDNA was synthesized from 300–600 ng of Total RNA using SuperScript III Reverse Transcriptase (Invitrogen) and 50 µM Oligo(dT)_12–18_ primer and incubated at 52°C for one hour followed by inactivation at 70°C for 15 minutes. qPCR primers were designed using Primer3 software [49] with the following criteria: primers 15–30 base pairs in length, annealing temperature 58–60°C and a 75–100 nt amplicon. *OnRPS3*-F, TGGTAGTGTCTGGCAAGCTC, *OnRPS3*-R, CGTAGTCATTGCATGGGTCT; *OnOr1*-F, CGGCGTCAGCACCATGA, *OnOr1*-R, TCTCCCATTGTTTGCAGAATG; *OnOr2*-F,GCTCTGAAGAAGCCAAGACC, *OnOr2*-R, CAAGTCCAGTGAAACCGTGA; *OnOr3*-F,GGCGCACCGCTCATATC, *OnOr3*-R, CCCAACGCTTTGATGGTGAT; *OnOr4*-F, CTGGTGACCCTGGAGATGAT, *OnOr4*-R, CAAATGCCTCGGATGTTTTAG; *OnOr5*-F, TCACGGTCGGCGTCACTA, *OnOr5*-R, TTCGCAAGAACATGAAGTAAGAAAA, *OnOr6*-F, AGAGACGGAAAAGCTGAAGG, and *OnOr6*-R, TATCCCCAACATGGTGTTCA. Each primer set was validated by calculating standard curves with 10× serial dilutions of template (three replicated wells for each template dose). The threshold cycle (CT) was plotted against the log of the template dilution and primers with slopes ranging from 3.1 to 3.5 were used (a slope of 3.3 represents 100% efficiency).

qPCR experiments were performed using 96 well plates (Bio-Rad, Hercules, CA), the IQ5 Real Time PCR Detection System (Bio-Rad) and IQ SYBR Green Supermix (Bio-Rad). Each 15 µl reaction was replicated in triplicate. Cycling conditions were as follows: 95°C for 1 minute, 40 cycles of 95°C for 10 seconds and 58°C for 1 minute, followed by melting temperature analysis: 95°C for 1 minute, 58C° for 1 minute and 67 cycles of 55–88C° for 10 seconds. Baseline cycle and threshold values were calculated automatically using default settings. No-template and no-reverse transcriptase controls were included in each experiment. As a final validation, qPCR products were cloned into TOPO pCR-4 and sequenced to ensure that the expected product was amplified. Expression levels of *OnOrs* 1–6 were calculated relative to the control gene, *OnRpS3*, using the 2^−ΔCT^ method [50].

### Preparation of Oocytes

*Xenopus laevis* frogs were purchased from Nasco (Fort Atkinson, WI). The care and use of *X. laevis* frogs in this study were approved by the University of Miami Animal Research Committee and meet the guidelines of the National Institutes of Health. Frogs were anesthetized by submersion in 0.1% 3-aminobenzoic acid ethyl ester, and oocytes were surgically removed. Oocytes were separated from follicle cells by treatment with collagenase B (Roche, Indianapolis, IN) for 2 h at room temperature.

### cRNA Injections

Capped cRNA encoding each candidate pheromone receptor was synthesized from linearized template DNA cloned in pGEMHE using mMessage mMachine kits (Ambion, Austin, TX). cRNAs were then injected into Stage V-VI *Xenopus* oocytes at a concentration of 25 ng/cRNA species/oocyte. Oocytes were incubated at 18°C in Barth's saline (in mM: 88 NaCl, 1 KCl, 2.4 NaHCO_3_, 0.3 CaNO_3_, 0.41 CaCl_2_, 0.82 MgSO_4_, 15 HEPES, pH 7.6, and 100 µg/ml amikacin) for 2–5 days prior to electrophysiological recording.

### Electrophysiology and Data Analysis

Pheromone-induced currents were measured under two-electrode voltage clamp using an automated parallel electrophysiology system (OpusExpress 6000A; Molecular Devices, Union City, CA). Micropipettes were filled with 3 M KCl and had resistances of 0.2–2.0 MΩ. The holding potential was −70 mV. Pheromones were perfused with ND96 (in mM: 96 NaCl, 2 KCl, 1 CaCl_2_, 1 MgCl_2_, 5 HEPES, pH 7.5). Pheromone stock solutions (1 M) were prepared in DMSO and stored at −20°C. On the day of each experiment, fresh dilutions were prepared in ND96. Unless otherwise noted, pheromones were diluted in ND96 and applied for 20 sec at a flow rate of 1.65 ml/min with extensive washing in ND96 (10 min at 4.6 ml/min) between applications. Pheromone compounds typically greater than 99% purity were purchased from Pherobank, Plant Research International B.V., Wageningen, The Netherlands. Current responses, filtered (4-pole, Bessel, low pass) at 20 Hz (−3 db) and sampled at 100 Hz, were captured and stored using OpusXpress 1.1 software (Molecular Devices). Initial analysis was done using Clampfit 9.1 software (Molecular Devices). Dose-response analysis was done using PRISM 4 software (GraphPad, San Diego, CA). Dose-response curves were fit according to the equation: 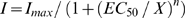, where *I* represents the current response at a given pheromone concentration, *X*; *I*_max_ is the maximal response; EC_50_ is the concentration of pheromone yielding a half-maximal response; and n is the apparent Hill coefficient. Relative efficacies of pheromones were normalized to the maximal response elicited by Z11–14:OAc.

## Supporting Information

Text S1 Pyrosequencing contigs, FASTA nucleotide file.
(0.03 MB DOC)

Figure S1 ClustalX alignment of OnOrs1 and 3–6.
(7.01 MB TIF)

Figure S2 Expression of OnOrs 1–6 in three different tissues of adult male and female moths: A) heads (with mouthparts); B) legs; and, C) abdomens. Gene expression, determined by real-time quantitative PCR with SYBR green, is reported relative to the reference gene OnRPS3. Expression was not detected in legs, no values are reported on the graph.
(0.26 MB TIF)

Table S1 Pyrosequencing contigs with homology to lepidopteran pheromone receptors.
(0.03 MB DOC)
