# Supplementary material for: Sex Pheromone Receptor Specificity in the European Corn Borer Moth, Ostrinia nubilalis
Source: PLoS One. 2010 Jan 13;5(1):e8685. doi: 10.1371/journal.pone.0008685 (PMC2801615; doi:10.1371/journal.pone.0008685)
Supplement: Text S1 — Pyrosequencing contigs, FASTA nucleotide file. (0.03 MB DOC) [file pone.0008685.s001.doc]

Supplementary Text S1. Pyrosequencing contigs, FASTA nucleotide file.

>contig02304(M4), OnOr1

acttcctgccgtgggagtgcatggacaccagcaaccggcggacggcgtgcatcatgctgcacaagatgcagtacaagatcagcctcaaggcgctggggctggcggccgtcggcgtcagcaccatgaccgggatattgaagacaacattttcatactacgcatttctgcaaacaatgggagattagcaaaatcgaactcgactttatttaatttgcttaaaaaaagattttgtcttgtaattgtcatgtttctatcttttatatttgtggcttctcagaataaacaatgataactagcaataaataagtagataatgaaaatattacttagttccatctgaattgtaacaactctttctataaattaata

>contig02899(M4), OnOr1

atttcggtggtcgcgatagtgcatagggtgactcngccgtaccacacgagcttcattgtcgtcggggagctttactacatatggatgcacatgcatgagttaacttttctcgatttgggacatatgattataacaacccttttaggaatactaacagcggtccgtagtatactaccacaattgcagaactatcataagttgctgctaaaatttataaatgtgatgcatttgatgcattccacaaataaaggaccgtattacaatcagatgaacgatactgtcgacaaagtttgttcatattatacaaaattttcgttaggattaatatttatttcgtgctcaatgtttaacgttgcaccattctgtaataacattgcaaacgtttttatttttaaaactgaaaactacactttggaattttctttatactaccaatttcccggaattgacccgaccgattacttcacaactacttcgatatacaatttctatctgtcgtacaattgcgctatgatggtgtctggactagatctaatattgtttttaataattttccaaataatcgggcacgtgtacatcctgagatacaaccttgagaactttccgtggccaaaaataaagtggtt

>contig02425(M4)

attttaaattaacgattctcaaagcaaacccaactcattggccaactttgaatttcaaacacttttcaaaagaggcacgagtgcaactctatataatatctctggtaatagatagatttacagatgaagtttcggcattgttcgggcccattttagccttgtaatatttgttt

>contig00228(M4), OnOr2

tgtacttcatctgtggggtcactgtcttggctgtaatgagttggataacaatcacgttcttcggagagtcagtccgcatgattgccaataaggaaaccaatgagaccctgacggaaccagctccgaggctgcctctgaagacgtggtaccccttcgatgctatgagtggcactatgtacgtcgtggcttttgtttaccaggtctactggctgttcttctccatggccatagcgaacctcatggacgtgatgttctgctcctggctcatcttcggtgcgagcagttgcagcatctgaaggccatcatgaagcccctgatggagctcagtgcttctttggatacgtacagacctaacactgctgaactgttccgtgcttcttctaccgaaaaatcagagaagatgccagacacagtggacatggacatccgtggcatctactccacgcagcaggacttcgggatgacccttcgaggagctggtggtagactccaaaacttcgggcagccgaaccctaacaatcccaatggattgacacagaagcaagagatgctggccagatctgccatcaagtattgggtggagaggcataagcacgtcgtgagattagtggcatccatcggggacacttatggcaccgctctgctgttccacatgttagtctccaccatcactctgactctcctggcttatcaggctacgaagatcaatgggataaacgtgtacgcgttcagcaccatcggatacctcagctacactctcggacaagtgtttcacttctgtatattcggaaaccggcttattgaagagagctcatcagtgatggaggcggcctactcctgccaatggtacgacggctctgaagaagccaagaccttcgtccagatcgtctgccagcagtgccagaaggcaatgagcatctctggggccaagttcttcacggtttcactggacttgttcgcttctgtacttggtgcagtggtgacttatttcatggttttggtacaactgaagtagaggatttgtataagttt

>contig03178(M3), OnOr2

tctggggccaagttcttcacggtttcactggacttgttcgcttctgtacttggtgcagtggtgacttatttcatggttttggtacaactgaagtagaggatttgtataagtttaaagagtgtaaaagtttctaaatagttttttgttttaattacattagaattagaatagttttttt

>contig01782(M4), OnOr3.

Aagtgcgcgtgcggctggcggagtgcatagaacatcacaaaattatcattcgattcacagatgaaatttcaattgttttcggccctattttagcctttaactacatgttccacatggtcggatgttgcttgctattgctggaatgttcagcgggaaaccaaataattcgttatgggcctctgacgactgtagtgtttggtcaacttattcaaatatcagttatgtttgagatgttaggtgctgagacggagaagctaaaggattcagcttacttcgtgccatgggagtgcatgaacaccagcaaccggcgcaccgctcatatcatgctgcacaagatgcaggacaaaatcagcatcaaagcgttgggtctggctgcggtcggagttaatactatgatggggattttgaagaccacgttttcgtactatgcattcctacaaacaatgaatgattaattttttgtagttatccagtaaatattataaaaaatatatactacaataaattgttcaacacgctataggcactacgcaccatgttatatttataagttcactcactacttattgcatgttatgtttaatcaccaagtgtaattaaattgtctaattgtaattaaaaatgaaagtatcaaacgtagaaaactcaaaagactt

>contig02068(M3), OnOr3

aagaaaactgcatcgtcgaaatgtttgacgcgaaggagaacgaagaagtgcgcgtgcggctggcggagtgcatagaacatcacaaaattatcattcgattcacagatgaaatttcaattgttttcgg

>contig00267(M3), OnOr6

aatggttacagctcttagaagtattctaccaaacttgcagacatacaattcattgctgtgtaaattcctacaggaatttcatttgatgcaccacgcttataaaggcgactattttgaagagatgaacaaaactgttgacaagatctcatcttattacacaaagtttagcacaataataatgtacttaggaaangntgntgttcaatatcctccaacgtacaacaacataagacataccttgatatcaaagactgaaaattattccatggaatattctgtatattttagttatcctggattcaacccgctcgaccactttgcaagtactaccatttataattgctacttatcatacaactgctcaacattgttgtgtgggtttgatttgttattgtttttgatgatatttcaaataattgggcacgtgtacattctgagacacaatctcgagaattttcagtcgcctaagaataaaattactctcaatttacggggagatgcattgagtacaaataacacgtgcacttatgaagtatttgacgcacaagaaaatgaagaagtgcgccttcaactggcggagtgcatagaacaccacaaaataataattggatttacagatgacctgtcagggctatttgggcctttattagccttcaattacttctttcatatgattgcctgttgtttgttgttactggaatgtacagaaggaagttatgatgcagtactacgatacggacctttgactatgatcgtttttggtcagctcatacagatgtcagttatgttcgagttgctggggtcagagacggaaaagctgaaggactccgtgtactatctgccatgggaggccatgaacaacagcaaccagcgaactgcttttataatgcttcacaaaatgcagtacaaaatcagtctcaaggcattaggactggcagcagttggcgtgaacaccatgttggggatattaaagactacgttttcatattatgcatttttacaaacaatgggagatagataatgagacgaaaaatgtatcattattacctacctacttcacaatttcacaatattttacaatatcttaattctttaccaatgattttcactttaatacttacttc

>contig00267(M3), OnOr6

aatggttacagctcttagaagtattctaccaaacttgcagacatacaattcattgctgtgtaaattcctacaggaatttcatttgatgcaccacgcttataaaggcgactattttgaagagatgaacaaaactgttgacaagatctcatcttattacacaaagtttagcacaataataatgtacttaggaaangntgntgttcaatatcctccaacgtacaacaacataagacataccttgatatcaaagactgaaaattattccatggaatattctgtatattttagttatcctggattcaacccgctcgaccactttgcaagtactaccatttataattgctacttatcatacaactgctcaacattgttgtgtgggtttgatttgttattgtttttgatgatatttcaaataattgggcacgtgtacattctgagacacaatctcgagaattttcagtcgcctaagaataaaattactctcaatttacggggagatgcattgagtacaaataacacgtgcacttatgaagtatttgacgcacaagaaaatgaagaagtgcgccttcaactggcggagtgcatagaacaccacaaaataataattggatttacagatgacctgtcagggctatttgggcctttattagccttcaattacttctttcatatgattgcctgttgtttgttgttactggaatgtacagaaggaagttatgatgcagtactacgatacggacctttgactatgatcgtttttggtcagctcatacagatgtcagttatgttcgagttgctggggtcagagacggaaaagctgaaggactccgtgtactatctgccatgggaggccatgaacaacagcaaccagcgaactgcttttataatgcttcacaaaatgcagtacaaaatcagtctcaaggcattaggactggcagcagttggcgtgaacaccatgttggggatattaaagactacgttttcatattatgcatttttacaaacaatgggagatagataatgagacgaaaaatgtatcattattacctacctacttcacaatttcacaatattttacaatatcttaattctttaccaatgattttcactttaatacttacttc

> Contig(M4), OnOr6

catacctgatatcaaagactgaaaattattccatggaatattctgtatattttagttatcctggattcaacccgctcgaccactttgcaagtactaccatttataattgctacttatcatacaactgctcaacattgttgtgtgggtttgatttgttattgtttttgatgatatttcaaataattgggcacgtgtacattctgagacacaatctcgagaattttcagtcgcctaagaataaaattactctcaatttacggggagatgcattgagtacaaataacacgtgcacttatgaagtatttgacgcacaagaaaatgaagaagtgcgccttcaactggcggagtgcatagaacaccacaaaataataattggatttacagatgacctgtcagggctatttgggcctttattagccttcaattacttctttcatatgattgcctgttgtttgttgttactggaatgtacagaaggaagttatgatgcagtactacgatacggacctt

>contig01209(M4),OnOr6

aaaaatgcagtacaaaatcagtctcaaggcattaggactggcagcagttggcgtgaacaccatgttggggatattaaagactacgttttcatattatgcatttttacaaacaatgggagatagataa tgagacgaaaaatgtatcattattacctacctacttcacaatttcacaatattttacaatatcttaattctttaccaatgattttcactttaatacttacttcgattttaagtacctacccacctatgatactgtttatcaaataaaattctgcatattttagatacttacctacctacttcaaatcatgtaggtattcatgactatagcatattgttgttttctttgtaagaatgtcttacctaagggttttacgaataaagttgtcgaacgaatatcacattaagcgagttcgttcgtatccttattatcggtaa
