# Supplementary figures and images for: Sex Pheromone Receptor Specificity in the European Corn Borer Moth, Ostrinia nubilalis
Source: PLoS One. 2010 Jan 13;5(1):e8685. doi: 10.1371/journal.pone.0008685 (PMC2801615; doi:10.1371/journal.pone.0008685)

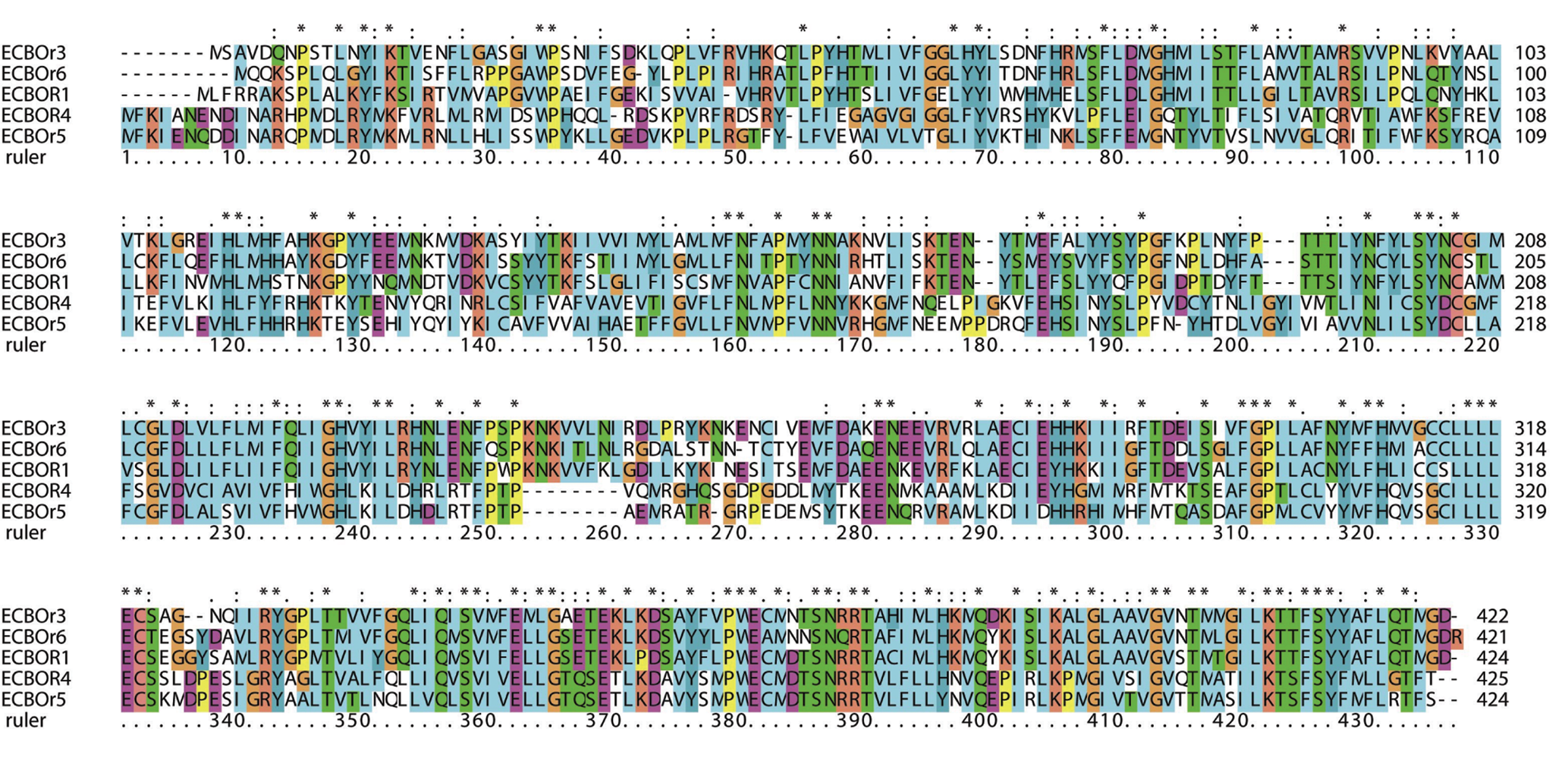

Supplement: Figure S1 — ClustalX alignment of OnOrs1 and 3–6. (7.01 MB TIF) [file pone.0008685.s002.tif]

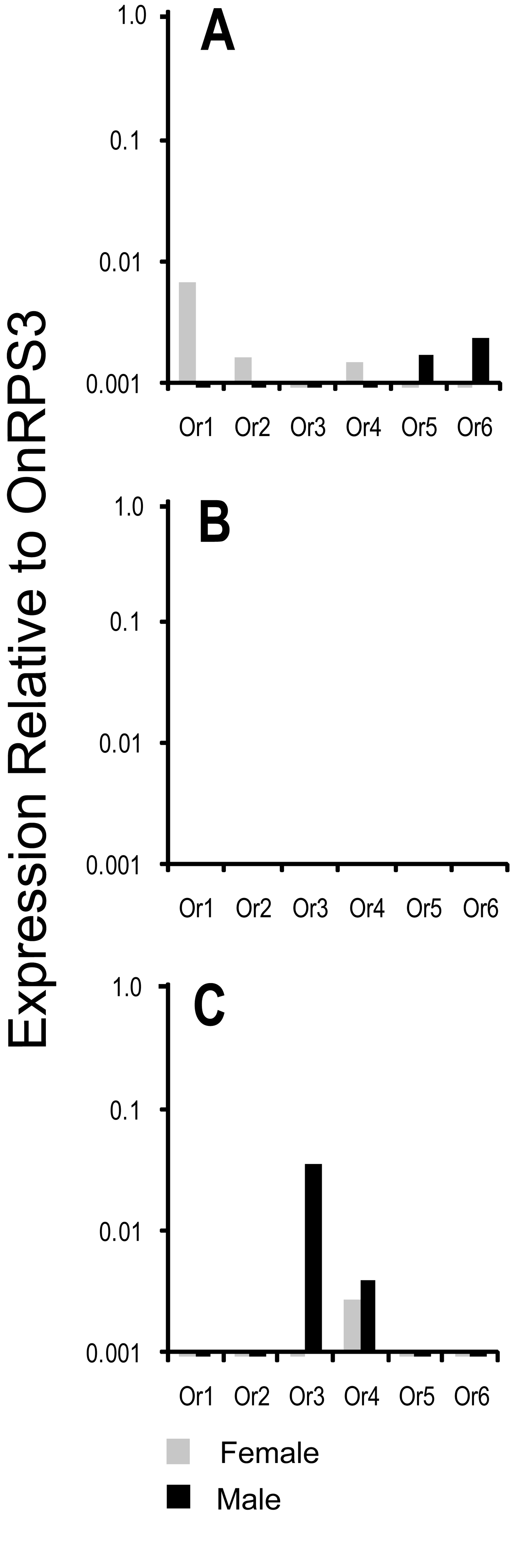

Supplement: Figure S2 — Expression of OnOrs 1–6 in three different tissues of adult male and female moths: A) heads (with mouthparts); B) legs; and, C) abdomens. Gene expression, determined by real-time quantitative PCR with SYBR green, is reported relative to the reference gene OnRPS3. Expression was not detected in legs, no values are reported on the graph. (0.26 MB TIF) [file pone.0008685.s003.tif]
