# Supplementary material for: Sex Pheromone Receptor Specificity in the European Corn Borer Moth, Ostrinia nubilalis
Source: PLoS One. 2010 Jan 13;5(1):e8685. doi: 10.1371/journal.pone.0008685 (PMC2801615; doi:10.1371/journal.pone.0008685)
Supplement: Table S1 — Pyrosequencing contigs with homology to lepidopteran pheromone receptors. (0.03 MB DOC) [file pone.0008685.s004.doc]

**Supplementary Table S2.** Pyrosequencing contigs with homology to lepidopteran pheromone receptors.

| **Receptor** | **C-terminus from**  **degenerate PCR** | **Contigs from pyrophosphate sequencing** | |
| --- | --- | --- | --- |
|  |  | **cDNA from mRNA** | **Amplified/normalized cDNA** |
| **OnOr1** | FJ385011.1 & FJ385012.1 | **c2425**, 173 nt, 5 reads, c2304, 369 nt, 12 reads  c2899, 626 nt, 11 reads | None |
| **OnOr2** | N/A | c228, 1032 nt, 62 reads | c3178, 178 nt, 6 reads |
| **OnOr3** | FJ385014.1 | c1782, 656 nt, 18 | **c2068**, 127 nt, 2 reads |
| **OnOr4** | FJ385013.1 | None | None |
| **OnOr5** | FJ385015.1 | None | None |
| **OnOr6** | none | **c1209**, 444 nt, 9 reads c2468, 510 nt, 14 reads | c267, 1124 nt, 21 reads |

Notes: Contigs **2425**, **1209** and **2068** were detected afterwards by using full length receptors for BLASTn searches. **c2425** is a short contig that includes 115 nt of intron sequence, **c2068** was the shortest contig and the majority of **c1209** is 3’UTR.
